# Proposing and developing a National Institute for Forensic Toxicology in Ireland - transformation through education

**DOI:** 10.1186/2193-1801-2-360

**Published:** 2013-07-30

**Authors:** William P Tormey

**Affiliations:** Biomedical Sciences, University of Ulster, Coleraine, BT52 1SA Northern Ireland UK

## Abstract

Toxicology for coroners is an important minority service whose quality is central to the validity and integrity of the death certification system. Multidisciplinary case conferences are routine practice in the major medical specialties. In conventional practice for the coroners’ courts, the dissecting pathologist is often relied upon to interpret toxicological data usually without specialist training. In Ireland the service is fragmented and there is at present no medically trained toxicologist directly involved in the direction or reporting of cases even where multiple drugs are found in blood at various levels and drug-drug interaction may be possible or likely. To encourage both medical professionals and political administration to confront the issues, the education literature on action research and critical incident methodology is reviewed to develop a strategy for change in this service. An example of action research being applied to medicine is used as an indicator that an action research template could be used to advocate for a new National Institute for Forensic Toxicology in Ireland. The main participant groups are identified and the development cycle outlined.

## Medicine and the coroner

Minority medical specialities are often sided-tracked, ignored and under-resourced. Forensic toxicology is one such area of practice in Ireland. The investigation pathway in forensic biochemistry begins at the site of death and continues through the autopsy process, the analytical laboratory and the construction of a report for a pathologist who subsequently distils the autopsy findings and conclusions for presentation to the coroner. Each discipline commands its own expertise. These include forensic pathologists at the site of a suspicious death; hospital doctors or general practitioners in conventional medicine; anatomic pathologists at autopsy, laboratory scientists in different disciplines; and pathologists collating the final report. The coroner is a government official usually either a lawyer or a doctor who is charged with determining the cause, time, and manner of death. It is an ancient office originating in England in 1194. The coroners’ court is a court of law. In Ireland, the coroners’ core function is to investigate sudden and unexplained deaths so that a death certificate can be issued. This is an important public service to the living and in particular to the next-of-kin and friends of the deceased.

The coroner service not only provides closure for those bereaved suddenly but also performs a wider public service by identifying matters of public interest that can have life/death consequences ( 2013a). The coroner can make public recommendations in this regard.

In medical specialties, multidisciplinary case conferences are conventional in hospital practice. Difficult cases can be dissected and differential diagnoses discussed. The original genre in this activity is the conventional clinicopathological conference. The New England Journal of Medicine offers the prototype case conference at the Massachusetts General Hospital on a weekly basis for many years. There is considerable variation amongst coroners in verdicts in the UK and consensus conferencing should be a routine service to coroners to try to decrease variation and increase accuracy (Tormey & Moore 2012a).

The quotation below features on the website of the Dublin District Coroner’s Court.

**“Show me the manner in which a nation cares for its dead, and I will measure with mathematical exactness the tender mercies of its peoples, their loyalty to high ideals and their regard for the laws of the land. William Ewart Gladstone”**

Accuracy in determining the cause of death is important for society and can influence decisions in public health policy.

The Victorian Institute for Forensic Medicine is the prototype (in my opinion) for forensic services ( 2013b). It covers medico-legal death investigations, clinical forensic medicine, forensic scientific services, disaster victim identification, donor tissue bank of Victoria and academic courses in association with Monash University.

For the Republic of Ireland, there are already forensic science laboratory services in the State Laboratory at Back Weston in Celbridge, Kildare and at the Forensic Science laboratory at the Phoenix Park in Dublin. There is a serious deficit in the provision of standard clinical biochemistry particularly involving vitreous samples and some poisons such as paraquat/diquat and cyanide. No medically trained doctor is involved in the selection of forensic toxicology investigations or in the reporting of results at present. Consequently, expert advice for the autopsy pathologist is haphazard. This unacceptable deviation from professional standards must be addressed and corrected.

### Can the literature on action research facilitate the planning and development of a new service?

Forensic toxicology is evidence based in that laboratory results are produced in accredited laboratories. This ensures a degree of certainty and reliability in the analytical results. It is conventional to appraise the evidence based on a hierarchy of characteristics. These are

1. Are the results valid?
2. Are the results significant?
3. Are the results generalisable?

The questions of significance and generalisability are highly relevant in the toxicology in coroners’ cases. Because the medical literature is voluminous and complex, experience across a wide medical spectrum is necessary to preserve high standards of evidence-based interpretation and diagnosis. The strength of medical evidence increases in validity from expert opinion, through case reports, cohort studies and randomised controlled trials, to systematic reviews with meta-analysis.

Action Research is a paradigm which can usefully be incorporated into the project. The concept originated with Kurt Lewin in 1934 and can be succinctly defined as “No action without research; no research without action” (Adelman 1 993). Action Research is appropriate to promote partnership working (Winter & Munn-Giddings 2001). Action Research should lead to change and that change should be incorporated into the research process itself. The value of Action Research in its inclusivity is the key to successful change development (McVicar et al. 2012). Reflection as a way of developing practices is also a constructive ingredient in the change process (Leitch & Day 2000). It is a matter of ‘do and review’.

### Participatory action research

Part of this theory is applicable to medicine. It involves groups examining the relationships between practices and between knowledge, identity, agency and practice. An objective is to transform both theory and practice and treats these as mutually dependent. The object is to change the world for the better. There is dissemination of ideas which is engagement and networking with others in the field and this is a crucial aspect of the method. There is a process of action, reflection and analysis followed by planning, further intervention, reflection and analysis (Locke et al. 2013).

#### Practitioner action research

‘Action research that is initiated by practitioners is engaged in for the purpose of professional or organisational development/learning’ (Anderson and Herr 2009). Many studies in education avoid exposing shortcomings in organisation and avoid issues of power, inequities or controversy. Among administrators, there is a tendency to conservatism (Anderson & Jones 2000). The problem in education is that administrators occupy a position of hierarchical power over teachers and students but are themselves vulnerable and answerable to board members or regional power structures.

In schools and in colleges, there is a need to look good. This imperative works against action research which might research problems and likely expose short-comings which could embarrass the institution. So organisations may be anti-learning and develop defensive strategies in the guise of supervision of staff development projects (Argyris & Schon 1974). In the interests of organisational improvement, there is a need to identify and subsequently overcome organizational defensiveness. The use of practitioner action research to facilitate organizational learning is an important method in encouraging change. It is the micro-examination of ‘business as usual’ that leads to practitioner and organisational learning and change. This is the opposite to defensive reflexes that protect institutions and individuals from critical analysis with the potential to expose some less-than-optimal practices.

#### Critical incident methodology

A critical incident is something that happens that requires and demands further investigation, thought and reflection. The analysis both facilitates and directs detailed examination of the current conventional practice and through reflection may suggest a methodological improvement.

The questions which should be addressed in ‘critical incidents’ include: – whose interests are served or denied by the actions taken? What conditions sustain or preserve the actions? What power relations are involved? What structural, organisational, and cultural factors keep alternatives from arising? (Smyth 1991).

In medical specialities, critical incidents may generate litigation, enquiries or more benignly, a published case report. Medical case reports can inform practice but in evidence-based medical practice, case reports are low in the hierarchy. But critical incidents buttressed by case reports are useful to suggest both action research and change.

Action research changes people’s understanding of their own practices and the conditions under which they practice. Action research is also a practice of doings and relationships. It may be *technical practical and critical* (*Kemmis*^2009^). As defined by Kemmis (2009), action research is a practice-changing practice and does apply to medicine.

##### Technical action research

The researcher aims to improve practice to improve the death certification for coroners. This may involve changing the way others work and the system organisation works. The researcher decides what is to be done and what should be changed. The researcher must interpret the data examined. In technical action research there is a one-way relationship with others involved in or affected by the research (Kemmis 2009).

##### Practical action research

The project is self-directed and others, other than the researcher, have a voice. The means and ends of the practice are questioned. The views and responses of others will direct the way things are explored but the fundamental questions and the end change direction will be instigated by the practitioner.

##### Critical action research

The research is undertaken collectively. The theoretical issues which drive change are : practices that are unsustainable which are based on contradictory or false ideas; practices which are morally or socially unsustainable by unreasonably excluding self-expression; practices that are ecologically and materially unsustainable or economically and personally unsustainable.

## Transformation in medicine

Detailed processes in an Accident and Emergency Department were examined critically leading to significant changes in practice that included the hiring of a patient advocate and greeters at the arrival foyer, better communication with patients by the doctors, room stocking in the mornings, meal tickets provided when there are long delays, improved triage through education and meetings with pharmacy and phlebotomy to clarify expectations and improve support (Eisenberg et al. 2006). Thus a qualitative action research study was used to improve and streamline the care pathways and gain insight to the problems. The paper is an example of action research applied to medicine in which the processes in an Accident and Emergency department could be studied and with appropriate leadership offer an improved and better experience for patients and staff.

## National institute for forensic toxicology – template for success

This can be achieved by networking the present existing facilities which include the State Laboratory, Beaumont Hospital Toxicology Department and the National Forensic Laboratory for Specimen Analysis and Research in Intoxicated Driving at University College Dublin as set out in two recent publications (Tormey & Moore 2013; 2012). When the potential participants are in multi-disciplines and currently work in different sites funded separately and associated with two government departments – Health and Justice – the principles of Action Research seem appropriate to lead to success. Practitioner Action Research is clearly involved and some critical incidents have fuelled the drive for change (Tormey 2012 Tormey & Moore ; 2012b).

## What now?

1. A questionnaire addressed to coroners to seek their views on what their ideal biochemical toxicology service would be.
2. A study of potential drug interactions in cases involving the Dublin Coroner’s Court in 2012.
3. A review and study of current uses of vitreous biochemistry in post-mortem diagnosis.
4. Ensure a suitable template for the investigation of medical diagnoses such as ketoacidosis, undiagnosed hyperosmolar coma, hypoglycaemia and other conditions.
5. Review of the performance of the state laboratory with regard to screening in blood, urine, vitreous and other matrices for the presence of a wide range of therapeutic drugs and compounds together with drugs of misuse and abuse as outlined in the annual reports of National Poisons Centres in the UK and Ireland(Alcohol and Drug Research Unit (2009).
6. Contact with the Forensic Medicine Services to agree the best service from their perspective.
7. Meetings with Department of Health, Justice, Forensic Pathologists and State Laboratory in the first instance.
8. The re-establishment of a biological service in forensic toxicology for coroners in a hospital.
9. Develop a team for medical clinical interpretation of data to advise the coronial process.
10. Affiliate to the Faculty of Pathology at the Royal College of Physicians of Ireland.

Contact has been made with the Irish Departments of Justice and Health; the Coroners Society; the State Forensic Pathologists; the State Laboratory; University College Dublin; Beaumont, Galway University, St James University and Cavan Hospitals in Ireland; and a team of chemical pathologists and anatomic pathologists. These contacts have been face to face to agree the general principles and the way forward as set out above.

The purpose of this paper is to add academic impetus and drive the project forward through the peer review process accepted internationally. The absence of medical direction in forensic toxicology in Ireland constitutes a ‘critical incident’ which will drive the action research.

## References

[CR1] (2013). Overview of the Coroner Service.

[CR2] (2013). Forensic services.

[CR3] Adelman C (1993). Kurt Lewin and the Origins of Action Research. Ed Action Res.

[CR4] (2009). 2009 National Report (2008 data) to the EMCDDA by the Reitox National Focal Point. Ireland: new developments; trends and in-depth information on selected issues.

[CR5] Anderson GL, Herr K, Noffke S, Somekh B (2009). Practitioner action research and educational leadership. The Sage handbook of educational action research.

[CR6] Anderson GL, Jones F (2000). Knowledge generation in educational administration from the inside-out: The promise and perils of site-based, administrator research’. Educ Administration Q.

[CR7] Argyris C, Schon D (1974). Theory in Practice: increasing professional effectiveness.

[CR8] Eisenberg EM, Baglia J, Pynes JE (2006). Transforming emergency medicine through narrative: qualitative action research at a community hospital. Health Commun.

[CR9] Kemmis S (2009). Action research as a practice-based practice. Educ Action Res.

[CR10] Leitch R, Day C (2000). Action research and reflective practice: towards a holistic view. Educ Action Res.

[CR11] Locke T, Alcorn N, O’Neill J (2013). Ethical issues in collaborative action research. Educ Action Res.

[CR12] McVicar A, Munn-Giddings C, Abu-Helil C (2012). Exploring the development of action research in nursing and social care in the UK: a comparative bibliometric review of action research designs in social work (2000–2010). Action Res.

[CR13] Smyth J (1991). Problematising teaching through a ‘critical’ approach to clinical supervision. Curric Inq.

[CR14] Tormey WP (2012). Cannabis misinterpretation and misadventure in a coroner’s court. Med Sci Law.

[CR15] Tormey WP, Moore TM (2012). Consensus conferencing in forensic toxicology for the coronial system. Medico-legal J.

[CR16] Tormey WP, Moore T (2012). Inconsistent coroner interpretation of cardiac death in the presence of cannabis in urine. Forensic Tox.

[CR17] Tormey WP, Moore T (2013). Poisonings and clinical toxicology: a template for Ireland. Ir J Med Sci.

[CR18] Tormey WP, O’Shea P (2012). Reporting biochemical toxicology to the coroner must be improved. Ir Med J.

[CR19] Winter R, Munn-Giddings C (2001). A handbook for action research in health and social care.

